# The calcium sensor OsCBL1 modulates nitrate signaling to regulate seedling growth in rice

**DOI:** 10.1371/journal.pone.0224962

**Published:** 2019-11-07

**Authors:** Jing Yang, Xiaolong Deng, Xiaoxin Wang, Jingzhang Wang, Shiyun Du, Yangsheng Li

**Affiliations:** 1 State Key Laboratory of Hybrid Rice, College of Life Sciences, Wuhan University, Wuhan, P. R. China; 2 Institute of Rice Research, Anhui Academy of Agricultural Sciences, Hefei, P. R. China; INRA, FRANCE

## Abstract

Nitrate signaling integrates and coordinates gene expression and plant growth; however, the underlying molecular mechanisms involved remain poorly understood. Our previous study revealed that rice calcineurin B-like protein 1 (OsCBL1) modulates lateral root elongation by affecting auxin biosynthesis. Here, we report that OsCBL1 also modulates nitrate signaling to regulate rice seedlings growth. Compared with wild-type seedlings, seedlings of *OsCBL1*-knockdown (*OsCBL1*-KD) plants showed a suppressed growth phenotype, which included reduced root and shoot fresh weights and shorter radicles, crown roots, and lateral roots, when grown in nitrogen-free conditions. Although the growth defects of *OsCBL1*-KD plants could be partially rescued by the addition of nitrate to the growth conditions, the nitrate uptake capability of the *OsCBL1*-KD plants did not differ from that of wild-type plants as assessed via nitrate content and ^15^NO_3_^−^ influx experiments. The nitrate-regulated expression of nitrate signal sentinel genes (Os*NRT2*.*1* and Os*NRT2*.*2*) was affected in the *OsCBL1*-KD plants under both long- and short-term nitrate treatments. Overall, our results showed a novel role for OsCBL1 in the regulation of nitrate signaling and nitrate-mediated rice growth.

## Introduction

Because they cannot escape from harsh environmental conditions like animals can, plants have evolved a sophisticated system to sense and adapt to changes in their surrounding environment, including nutrient variations. Nitrate (NO_3_^−^) is a major nitrogen source for most land plants and is known to be a dual-function molecule.

NO_3_^−^ is not only a nutrient source but also a signaling molecule at the center of communication between plant genetic programs and the environment. The NO_3_^−^ signaling has both long- and short-term effects. The long-term effects are important for triggering different physiological events involving plant growth affected by NO_3_^−^, including seed germination, major root and leaf growth, and the transition to the reproductive stage [1–5]. The short-term effects involve the regulation of gene expression after a short period of exposure to NO_3_^−^. At the molecular level, NO_3_^−^ application can strongly and rapidly affect gene expression, which is thought to be crucial for the ability of plants to sense nutrient conditions and alter their growth process [6]. These rapid and often transient transcriptional inductions in response to NO_3_^−^ are the short-term effects of NO_3_^−^ signaling and are also referred to as the primary nitrate response (PNR) [7].

The PNR can occur in nitrate reductase (NR)-null mutants, which means the NO_3_^−^ itself triggers the induction rather than its downstream assimilation products [8]. The PNR can also occur in the presence of protein synthesis inhibitors, showing that it does not require *de novo* protein synthesis [9, 10]. In *Arabidopsis*, many NO_3_^−^ transport and assimilation genes, such as *NRT2*.*1*, *CHL1/NRT1*.*1*, *NIA1*, *NIA2*, and *NIR*, serve as sentinels for the PNR [2, 11]. One of the first genes found to affect the PNR was *CIPK8*, which encodes a calcineurin B-like (CBL)-interacting kinase that is rapidly induced by NO_3_^−^ and differentially regulated in *CHL1/NRT1*.*1* NO_3_^−^ transceptor mutants (*chl1–5*) (9). Several PNR sentinels, including *NRT2*.*1*, *CHL1*/*NRT1*.*1*, *NIA1*, *NIA2* and *NiR*, reduce the magnitude of induction in *cipk*8 mutants exposed to high-nitrate conditions, suggesting that CIPK8 is a positive regulator during the low-affinity phase of the PNR [9]. Expression of the *CIPK23* gene is also transiently induced by NO_3_^−^ and acts as a negative regulator of the PNR in the both low- and high- affinity phases [11].

The regulatory effect of CBL-interacting protein kinases (CIPKs) on the PNR indicates that a Ca^2+^ signal is involved in the perception and transmission of NO_3_^−^ signaling. Moreover, recent evidence has shown that nitrate treatment increases cytoplasmic Ca^2+^ concentrations and activates Ca^2+^-sensor protein kinases (CPKs), which phosphorylate NLP transcription factors to regulate nitrate-responsive gene expression [2, 12]. As another kind of Ca^2+^ sensor, CBLs contain four EF-hand domains for Ca^2+^ binding and specifically interact and activate CIPKs to transduce calcium signals [13]. CBL7 is involved in the regulation of the low-NO_3_^−^ response in *Arabidopsis* [14]. Whether and how CBLs play roles in the regulation of NO_3_^−^ signaling remain unclear. In the present work, we provide evidence that OsCBL1 is involved in both long- and short-term NO_3_^−^ signaling regulation, which in turn modulates rice seedling growth.

## Materials and methods

### Plant materials and growth conditions

Experiments were performed with wild-type (WT) rice (ShijinB) and transgenic *OsCBL1*-knockdown (*OsCBL1*-KD) plants reported in our previous study [15]. Seeds of the WT and knockdown plants were surface sterilized with 5% (v/v) NaClO at room temperature for 30 min and then rinsed with double-distilled water. The seeds were subsequently germinated in water at 30°C for 2 days prior to placement in 5-L vessels that contained H_2_O or solutions of different NaNO_3_ concentrations for an additional 7 days. The plants were grown in a growth chamber at 26/22°C and under a 16 /8-h light/dark photoperiod. To evaluate the PNR, 7-day-old plants growing in H_2_O were treated with different concentrations of NaNO_3_ or NaCl for the indicated time.

### Gene expression analysis

Total RNA was isolated from the root using TRIzol reagent (Invitrogen, Cat no. 15596026). An amount of ~ 2 μg of total RNA was extracted and treated with RNase-free DNase I before it was reverse transcribed to cDNA. Quantitative real-time PCR (qRT-PCR) was performed in a Bio-Rad CFX96^TM^ Real-time System (Bio-Rad, http://www.bio-rad.com) in conjunction with SYBR Green real-time PCR Master Mix. Data analysis was performed with Bio-Rad CFX Manager 3.0 software. The relative expression of target genes was normalized using the housekeeping gene *Actin* and *EF-1a*. The primers used for qRT-PCR are listed in S1 Table.

### Measurement of NO_3_^−^ content and ^15^N influx

Seven-day-old plants were used to measure the NO_3_^−^ content and ^15^N influx. The total amount of NO_3_^−^ was measured as previously described [16]. The shoots and roots of 7-day-old seedlings grown under different NO_3_^−^ concentrations were collected. Approximately 0.1 g of fresh tissue samples was then ground to powder in liquid nitrogen, suspended in 1 mL of water and incubated at 45°C for 1 hour. The supernatant was collected after centrifuging at 10000 g for 15 min at 4°C and sequentially reacted with salicylic acid–H_2_SO_4_ for 20 min. After adding 2 mL of 2 M NaOH, the solution was measured at a 410-nm wavelength, and then the NO_3_^−^ concentration was calculated according to a standard curve.

A ^15^N-influx assay was performed with ^15^N-labeled NaNO_3_ (98% atom ^15^N-NaNO_3_, Sigma-Aldrich). Seedlings were grown in H_2_O for 7 days and then treated with 0.2 or 2 mM ^15^N-NaNO_3_ for 30 min. The seedlings were then transferred to H_2_O for 3 min and treated with 0.1 mM CaSO_4_ for 1 min to remove the ^15^N-Na NO_3_^−^ from the root surfaces. The roots were subsequently collected and dried at 75°C. Finally, the roots were ground, and the ^15^N content was determined using a Vario ISOTOPE cube analyzer (Elementar Analysensysteme, https://www.elementar.de/en.html) following the manufacturer’s instructions.

### Phenotypic characterization

Root images were collected using a Canon600D camera. The lengths of the radicle, crown roots, and lateral roots (near the base of the radicle, 0.5–2 cm from the seed) were measured using ImageJ software (http://imagej.nih.gov.ij/).

## Results and discussion

### The inhibited-growth phenotype of *OsCBL1*-knockdown plants can be partially rescued by NO_3_^−^

**Fig 1:**
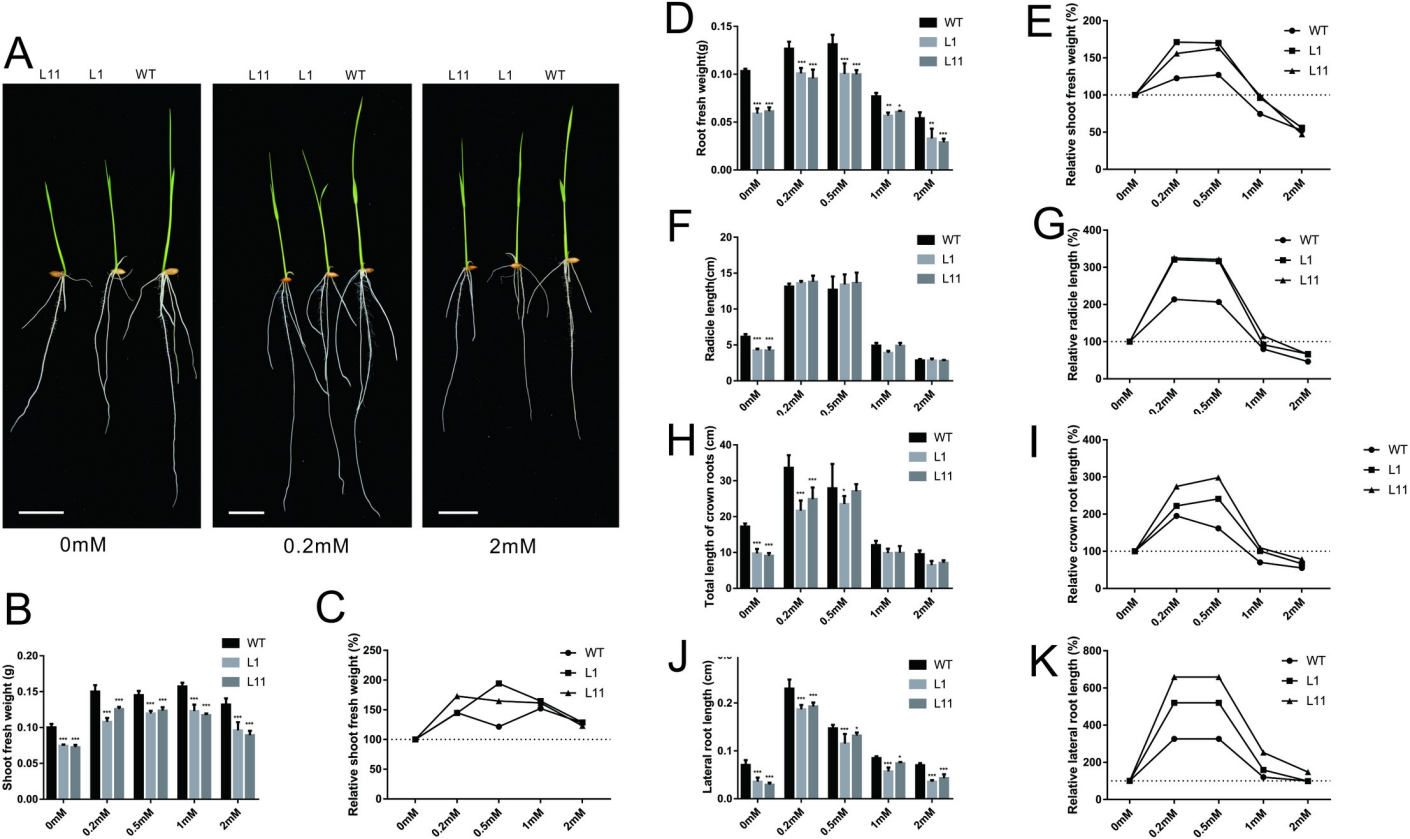
The inhibited-growth phenotype of *OsCBL1* knockdown plants can be partially rescued by NO_3_^−^. (A): Phenotypic assay of *OsCBL1*-KD under different NO_3_^−^ concentrations for 7 days. The statistic data are shown in S1 Fig. Fresh weight and root length under different NO_3_^−^ concentration (B, D, F, H, J) and the promotion or suppression effects of different NO_3_^−^ concentrations (C, E, G, I, K). Scale bar = 2 cm. The error bars represent ± SDs. (A) and (B–K) display two experimental replications. *, p < 0.05, **, p < 0.01, and ***, p < 0.001 compared to the WT (t test).

Our previous study showed that decreasing the expression of *OsCBL1* (i.e., *OsCBL1*-KD) inhibited the growth of rice roots under 1/2-strength Murashige and Skoog (MS) medium growth conditions [15]. Root growth is inextricably linked to nutrient elements. The *CBL1* gene has been reported to be involved in the uptake of K^+^ and NH_4_^+^ in *Arabidopsis* [17, 18]; furthermore, OsCBL1 localizes to the plasma membrane, and CBL1 is also involved in the regulation of K^+^ uptake in rice [19]. To further study how OsCBL1 participates in the regulation of rice growth and development and whether the regulation is related to the uptake of nutrient elements, we compared the growth of WT and *OsCBL1*-KD plants in H_2_O and in solution with different concentrations of NO_3_^−^. When the plants were grown in water, the growth of the *OsCBL1*-KD plants was significantly inhibited compared with that of the WT plants; when 0.2–2 mM NO_3_^−^ was supplied, the growth difference between *OsCBL1*-KD and WT was partially reduced (Fig 1 and S1 Fig). Low NO_3_^−^ concentrations (0.2–0.5 mM) significantly promoted the growth of rice seedlings, and the growth was more pronounced for *OsCBL1*-KD than for WT (Fig 1B–1K). High NO_3_^−^ concentrations (1–2 mM) suppressed the growth of WT seedlings, but this effect was weaker for *OsCBL1*-KD than for WT under 1 mM NO_3_^−^ conditions. Therefore, compared with the WT plants, the *OsCBL1*-KD plants were more sensitive to the stimulatory effects of low NO_3_^−^ but were insensitive to the inhibitory effects of high NO_3_^−^. These results indicate that in rice, OsCBL1 plays an important role in self-development programs and in the regulatory effects of NO_3_^−^ on rice growth.

### The growth inhibition of *OsCBL1* knockdown plants is not associated with NO_3_^−^ uptake or transport

**Fig 2:**
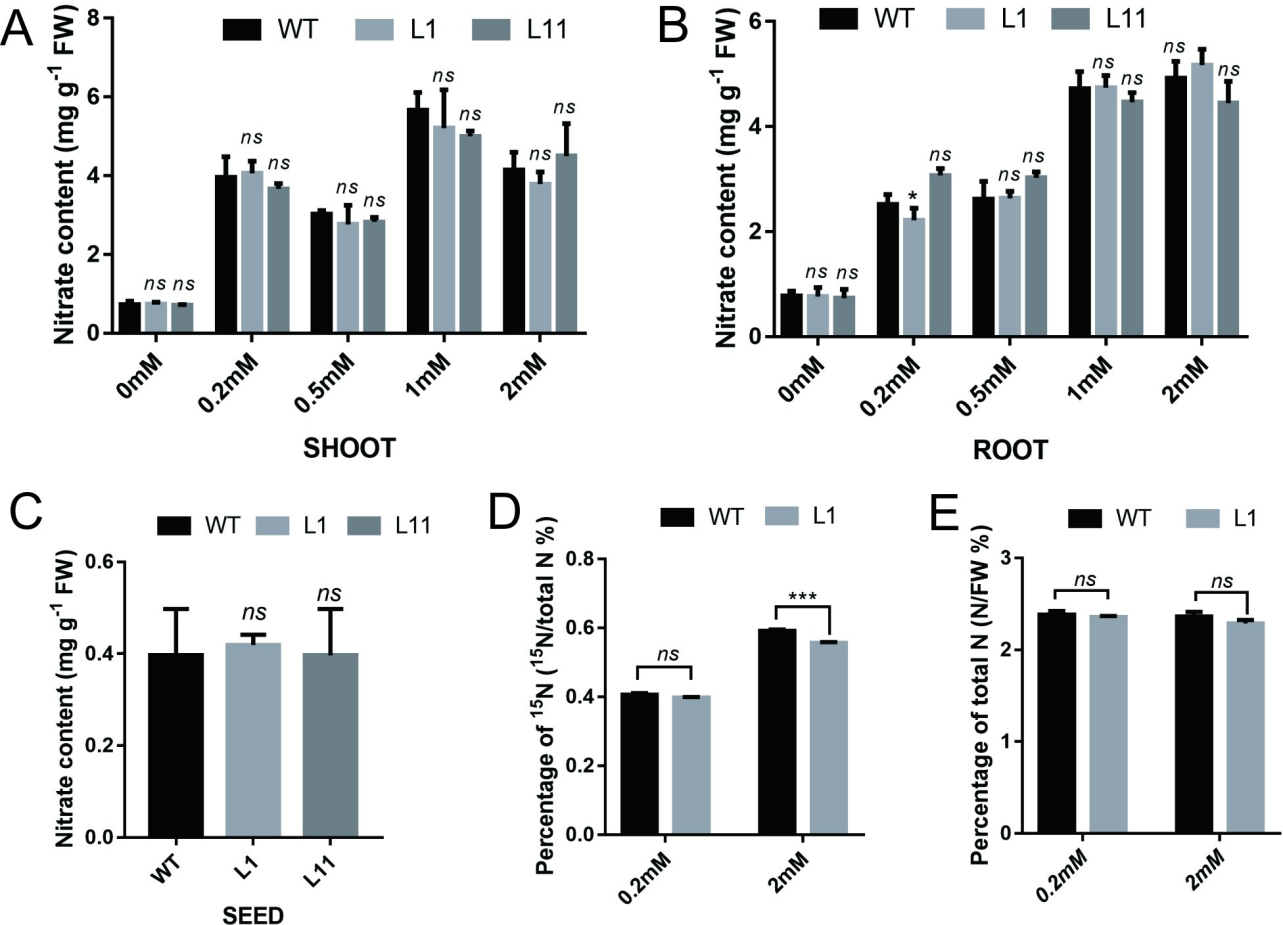
The effects of *OsCBL1* knockdown on NO_3_^−^ uptake and transport. (A) Shoot and (B) root NO_3_^−^ contents in OsCBL1-knockdown and WT plants under different NO_3_^−^ concentration for 7 days. (C) NO_3_^−^ content in the seeds of *OsCBL1*-knockdown and WT plants. (D) ^15^N content and (E) total nitrogen content in 7-day-old seedlings that were transferred from H_2_O conditions to solutions containing 0.2 mM or 2 mM ^15^NO_3_^-^ for 30 min. The error bars represent ± SDs. *, p < 0.05, ***, p < 0.01, and *ns*, not significant compared to the WT (t test).

To investigate how OsCBL1 influences the regulatory effects of NO_3_^−^ on rice growth, we first analyzed the NO_3_^−^ content in 7-day-old WT and *OsCBL1*-KD plants under different growth conditions. There were no significant differences in the content of NO_3_^−^ in the roots or shoots between the WT and *OsCBL1*-KD plants (Fig 2A and 2B); the NO_3_^−^ content in seeds also did not differ (Fig 2C). Using ^15^N-labeled NO_3_^−^, we then compared the uptake of NO_3_^−^. The WT plants absorbed slightly more ^15^ NO_3_^−^ than did the *OsCBL1*-KD plants when supplied with 2 mM ^15^NO_3_^−^ for 30 min, but no significant difference was detected when the plants were supplied with 0.2 mM ^15^ NO_3_^−^ (Fig 2D). Similar to what occurred for the NO_3_^−^ content, there was no significant difference in nitrogen content between the WT and *OsCBL1*-KD plants after ^15^NO_3_^−^ treatment (Fig 2E). These results indicated that the growth difference between the WT and *OsCBL1*-KD plants was not due to the difference in NO_3_^−^ uptake capability or NO_3_^−^ content.

### OsCBL1 affects the expression of NO_3_^−^ transport-related genes under different NO_3_^−^ conditions

**Fig 3:**
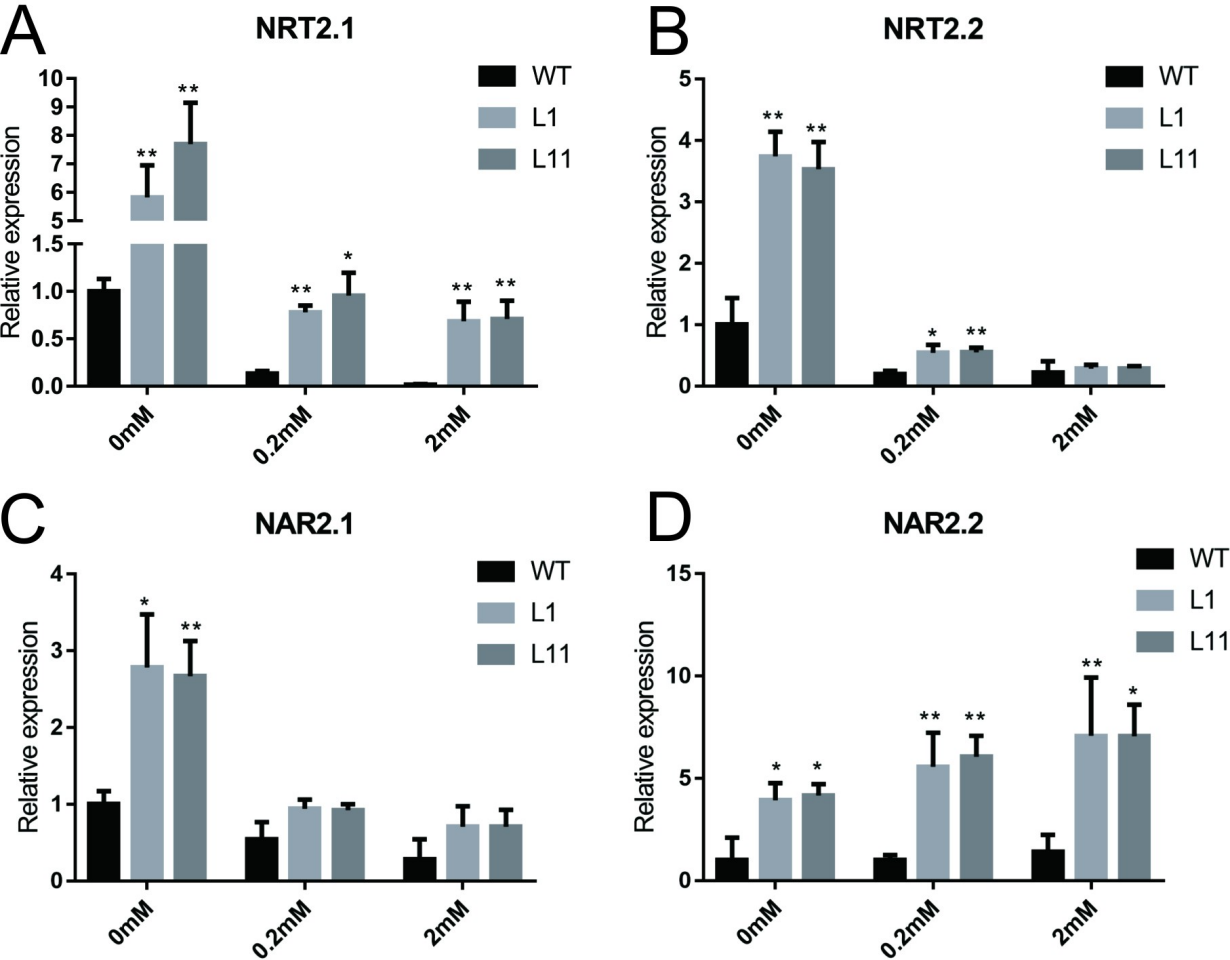
The effect of *OsCBL1*-knockdown on the expression level of NO_3_^−^-transport-related genes. Quantitative PCR analysis of the expression of two NO_3_^−^ transporter genes, *OsNRT2*.*1* (A) and *OsNRT2*.*2* (B), and two NO_3_^−^ transport-associated genes, *OsNAR2*.*1* (C) and *OsNAR2*.*2* (D). *OsCBL1*-knockdown and WT seedlings were grown under different NO_3_^−^ concentrations and gene expression levels in the roots were measured. The relative expression level was normalized to that in WT plants under 0 mM NO_3_^−^ concentration. The error bar represent ± SDs. *, p < 0.05 and **, p < 0.01 compared to the WT (t test).

In addition to being an essential nutrient, NO_3_^−^ acts as a signaling molecule to regulate gene expression. NO_3_^−^ signaling is at the center of communication between plant genetic programs and the environment and regulates plant growth, development and stress responses [20]. Many NO_3_^−^ transport- and assimilation-related genes have also been found to be involved in NO_3_^−^ signaling. To further investigate how NO_3_^−^ affects the growth of WT and *OsCBL1*-KD plants under different growth conditions, we evaluated the expression of some NO_3_^−^ transport-related genes (*OsNRT2*.*1*, *OsNRT2*.*2*, *OsNAR2*.*1*, and *OsNAR2*.*2*) under different growth conditions. The results showed that with the addition of NO_3_^−^, the expression of *OsNRT2*.*1*, *OsNRT2*.*2*, and *OsNAR2*.*1* decreased in both the WT and *OsCBL1*-KD plants (Fig 3A–3C), suggesting that the expression of these genes was induced by nitrogen starvation, similar to the results for nitrate transporter genes (*AtNRT2*.*1*, *AtNRT2*.*4*, *AtNRT2*.*5*) in *Arabidopsis* [14, 21, 22]. Under conditions of no and low NO_3_^−^ content, the expressions of *NRT*s and *NAR*s was higher in the *OsCBL1*-KD plants than in the WT plants (Fig 3A–3D), indicating the presence of altered NO_3_^−^ sensing in the *OsCBL1*-KD mutant. Considering that the NO_3_^−^ content in both the WT and *OsCBL1*-KD plants increased after NO_3_^−^ addition, and the lack of significant difference between WT and CBL1-KD plants (Fig 2A and 2B), these results indicate that the difference in the expression of these genes did not directly affect NO_3_^−^ uptake or translocation but may have affected the sensing and/or transmission of NO_3_^−^ signal, subsequently regulating rice growth. Compared with WT plants, the *OsCBL1*-KD plants in the same NO_3_^−^ conditions were not more NO_3_^−^ starved but seemed to respond more intensely to nitrogen starvation signals. Therefore, OsCBL1 likely plays an important role in signaling pathways involved in intracellular NO_3_^−^ perception.

### OsCBL1 regulates the primary nitrate response

**Fig 4:**
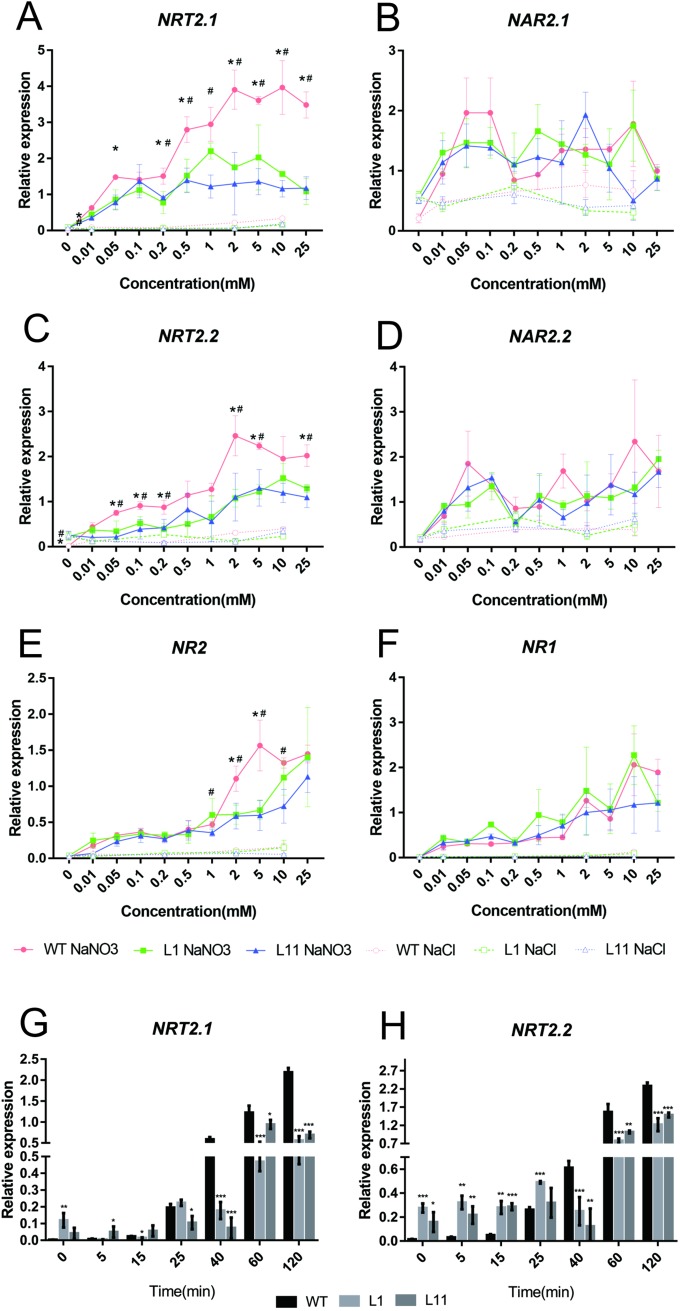
Primary nitrate response in *OsCBL1*-knockdown plants. Quantitative PCR analysis of the NO_3_^−^-induced expression of two NO_3_^−^ transporter genes *(OsNRT2*.*1*(A) and *OsNRT2*.*2*(C)), two NO_3_^−^ transport-associated genes (*OsNAR2*.*1*(B) and *OsNAR2*.*2*(D)), and two NO_3_^−^ assimilation genes (*OsNR1*(E) and *OsNR2*(F)). OsCBL1-knockdown and WT plants were grown in H_2_O for 7 days and then exposed to solutions of different NaNO_3_ or NaCl (control) concentrations for 2 hours. *, significant difference (p < 0.05) between the WT and L1 knockdown line; #, significant difference (p < 0.05) between the WT and L11 knockdown line. Quantitative PCR analysis of the expression levels of *OsNRT2*.*1*(G) and *OsNRT2*.*2*(H) induced by 2 mM NaNO_3_. *, p < 0.05, **, p < 0.01, and ***, p < 0.001 compared to the WT (t test).

As a sentinel for PNR, *AtNRT2*.*1* is induced not only by nitrogen starvation but also by short-term NO_3_^−^ treatment [7]. To further confirm that the NO_3_^−^ signaling changed in *OsCBL1*-KD plants, the expression of six NO_3_^−^ induced genes was analyzed in *OsCBL1*-KD plants to determine whether OsCBL1 is involved in the regulation of the PNR. These genes included two NO_3_^−^ uptake transporter genes, *OsNRT2*.*1* and *OsNRT2*.*2*, and their partners, *OsNAR2*.*1* and *OsNAR2*.*2*, as well as two NO_3_^−^ assimilation genes, *OsNR1* and *OsNR*2. Wild-type and *OsCBL1*-KD plants were grown in H_2_O for 7 days and then were exposed to different concentrations of nitrate solution. The expression levels of all six genes were significantly induced by NO_3_^−^ in both the WT and *OsCBL1*-KD plants. The magnitude of induction of *OsNRT2*.*1*, *OsNRT2*.*2*, and *OsNR2* was significantly reduced in *OsCBL1*-KD plants compared with WT plants under NO_3_^−^ induction (Fig 4A, 4C and 4E), while the expressions levels of *OsNAR2*.*1*, *OsNAR2*.2, and *OsNR1* were similar between the WT and *OsCBL1*-KD plants (Fig 4B, 4D and 4F). A decrease in the NO_3_^−^-induced expression of *OsNRT2*.*1* and *OsNRT2*.*2* occurred under both low and high NO_3_^−^ concentrations, while the expression of *OsNR2* was repressed only under high nitrate concentration (Fig 4A, 4C and 4E). These data suggest that the existence of the PNR pathways that either involve or do not involve OsCBL1. We further surveyed the time course of the expression of *OsNRT2*.*1* and *OsNRT2*.*2* under 2 mM NO_3_^−^ concentrations. Although the expression of the two genes was relatively high in OsCBL1-KD under nitrogen-free conditions, the expression increased more quickly and intensely in the WT plants under NO_3_^−^ treatment (Fig 4G and 4H). The expression of AtNRT2.1 in plants growing under high-N condition is inhibited [7] but is induced when exposed to nitrate for a short period of time regardless of whether plants grow under N-sufficient or N-deficient conditions [23].

These different regulatory activities indicate that there are different regulatory pathways between long-term and short-term nitrate signaling. Many genes have been characterized to regulate the expression of AtNRT2.1, such as *NLP6*, *NLP7*, *LBD37*/*38*/*39*, and *NIGT1*, which are involved in short-term nitrate signaling and *NLP7*, *TGA1*/*4*, and *HIN9*/*IWS1*, which are involved in long-term nitrate signaling [24]. Our results indicate that OsCBL1 is involved in both long- and short-term nitrate signaling and plays different roles in the regulation of OsNRT2.1 and OsNRT2.2 expression.

In Arabidopsis, two CBL-interacting protein kinases, CIPK8 and CIPK23, are involved in PNR regulation. The *CIPK8* gene is rapidly induced by NO_3_^−^, and CIPK8 acts as a positive regulator in PNR because the induction of several PNR sentinel genes by NO_3_^−^ is reduced in the *cipk8* mutant under high NO_3_^−^ concentrations [9]. The *CIPK23* gene is also transiently induced by NO_3_^−^, and the induction of *NRT2*.*1* by NO_3_^−^ is higher in the *cipk23* mutant than in WT plants at both high and low NO_3_^−^ concentrations [11]. The *OsCBL1* gene was not induced by NO_3_^−^ under long- or short-term treatment (S2 Fig), and its product differentially regulated the expression of different PNR marker genes depending on the NO_3_^−^ concentration (Fig 4). These results suggest that OsCBL1 may function as a converter that accepts different Ca^2+^ signals induced by different NO_3_^−^ concentrations and transduces Ca^2+^ signals downstream by activating different OsCIPKs and regulating gene expression.

A recent study revealed the function of Ca^2+^ sensor CPKs to be master regulators that regulate NO_3_^−^-activated signaling [2]. Here, we revealed the role of another type of Ca^2+^ sensor CBL in NO_3_^−^ signaling. Considering that CIPK is also involved in the regulation of NO_3_^−^ signaling [9, 11], the CBL–CIPK pathway should be another NO_3_^−^-coupled Ca^2+^ signaling mechanism that regulates the plant nutrient-growth network. The complex interaction between CBL and CIPK members indicates that the CBL–CIPK module might play an important role in relaying NO_3_^−^ signaling specifically to downstream targets. Future studies are likely to clarify how CBLs sense distinct Ca^2+^ signatures caused by nutrient signaling and identify targets of CIPKs, such as channels, transporters, transcription factors and other regulators involved in all aspects of nutrient-mediated growth regulation in plants.

## Supporting information

S1 Fig The root phenotype of WT and *OsCBL1*-knockdown plants under different nitrate concentration. Radicle (A) and crown root (B) length of 7-day-old plants were measured grown under different nitrate concentrations. *, p < 0.05, **, p < 0.01and ***, p < 0.001 compared to the WT (t test).
(TIF)

S2 Fig The expression pattern of *OsCBL1* under different nitrate treatment. (A) The relative expression levels of *OsCBL1* in 7-day-old WT plants grew under different NaNO_3_ concentrations. (B) The relative expression levels of *OsCBL1* in WT plants which grew under non-nutritional condition for 7 days and then were treated by different NaNO_3_ or NaCl (control) concentration solution for 2 hours.
(TIF)

S1 Table Primer sequences used in this study. (DOCX)
