# Supplementary figures and images for: The calcium sensor OsCBL1 modulates nitrate signaling to regulate seedling growth in rice
Source: PLoS One. 2019 Nov 7;14(11):e0224962. doi: 10.1371/journal.pone.0224962 (PMC6837758; doi:10.1371/journal.pone.0224962)

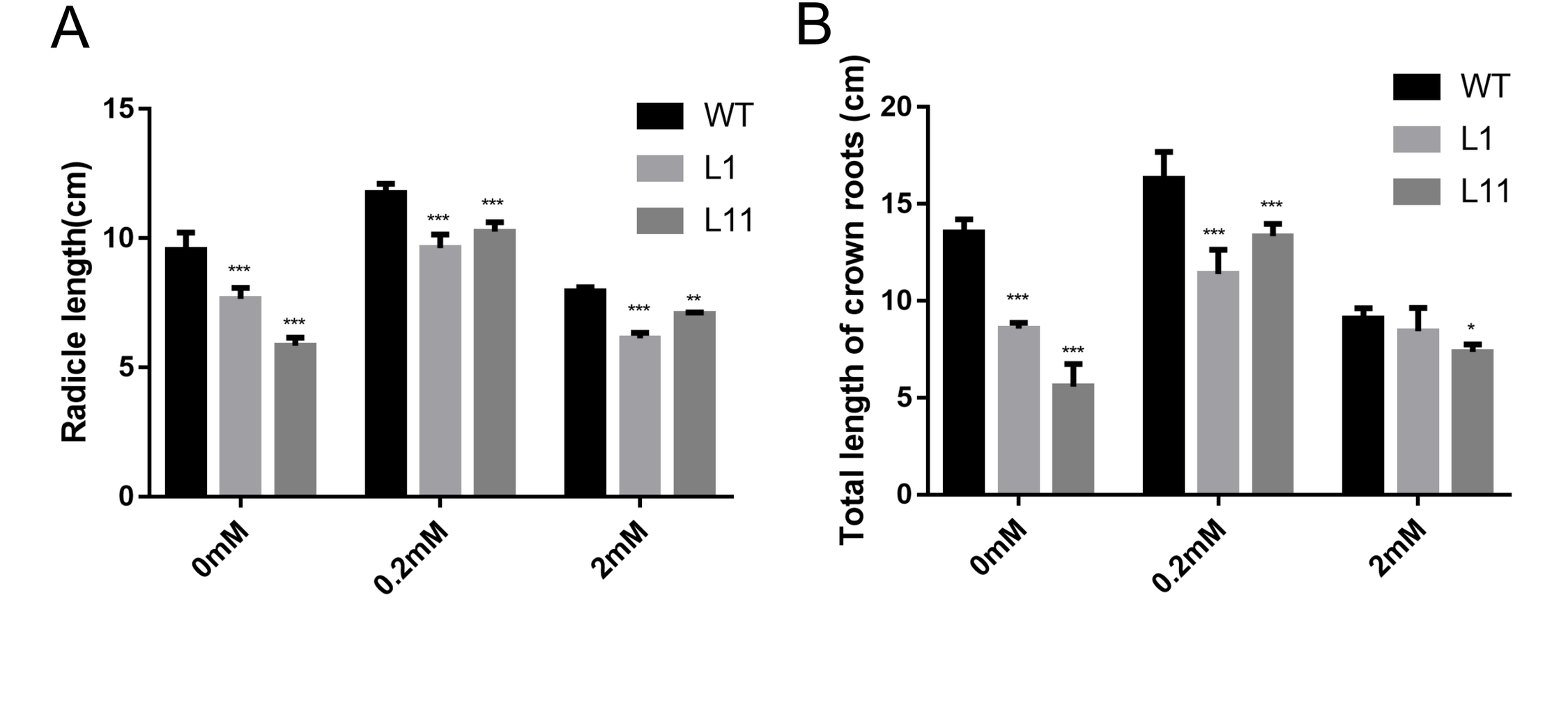

Supplement: S1 Fig — Radicle (A) and crown root (B) length of 7-day-old plants were measured grown under different nitrate concentrations. *, p < 0.05, **, p < 0.01and ***, p < 0.001 compared to the WT (t test). (TIF) [file pone.0224962.s001.tif]

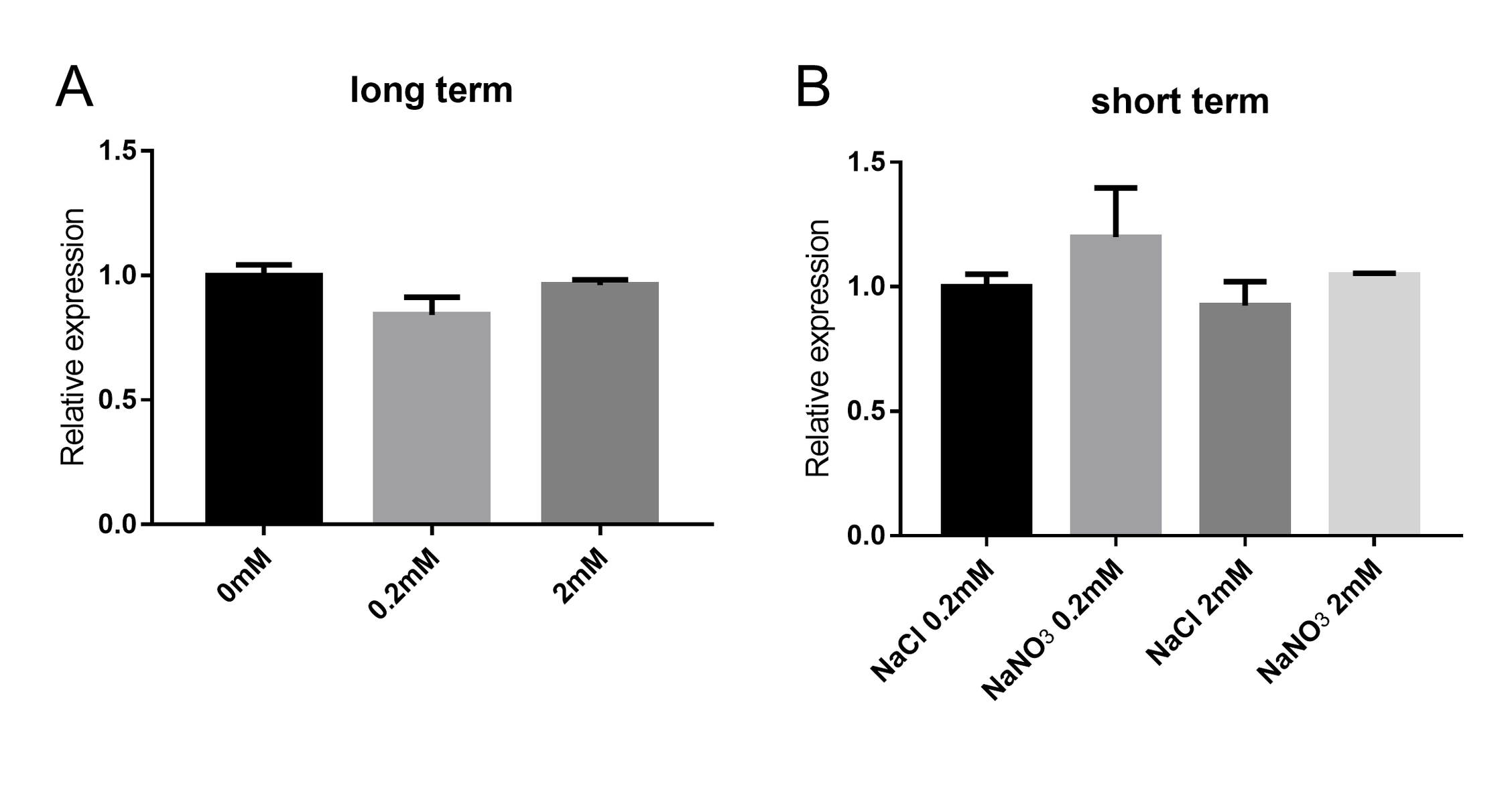

Supplement: S2 Fig — (A) The relative expression levels of OsCBL1 in 7-day-old WT plants grew under different NaNO3 concentrations. (B) The relative expression levels of OsCBL1 in WT plants which grew under non-nutritional condition for 7 days and then were treated by different NaNO3 or NaCl (control) concentration solution for 2 hours. (TIF) [file pone.0224962.s002.tif]
