# Supplementary material for: The calcium sensor OsCBL1 modulates nitrate signaling to regulate seedling growth in rice
Source: PLoS One. 2019 Nov 7;14(11):e0224962. doi: 10.1371/journal.pone.0224962 (PMC6837758; doi:10.1371/journal.pone.0224962)
Supplement: S1 Table — (DOCX) [file pone.0224962.s003.docx]

**S1 Table** Primer sequences used in this study

| primer name | sequence (5'-3') |
| --- | --- |
| Actin1-F | GGAAGTACAGTGTCTGGATTGGAG |
| Actin1-R | TCTTGGCTTAGCATTCTTGGGT |
| EF-1α-F | GCTGCTGCAACAAGATGGATG |
| EF-1α-R | CAGAGATGGGAACGAAGGGAA |
| OsCBL1-F | GTCGCACAGGATATTAGC |
| OsCBL1-R | TACGGGGACAAGGATAAG |
| OsNRT2.1-F | GTTCGACTCGATGATAACAC |
| OsNRT2.1-R | GGTATAAATGCCTCTCCCTTA |
| OsNRT2.2-F | CTGTTGAACGTTTTGTTACC |
| OsNRT2.2-R | GAAGCAAGTAATACAAAGGC |
| OsNAR2.1-F | GTGATGTTCTCCTCGGATA |
| OsNAR2.1-R | TTGAGTTACAGCAACCAATT |
| OsNAR2.2-F | TTGAGTTACAGCAACCAATT |
| OsNAR2.2-R | CGTTGGTTTTGTAGGTTGA |
| OsNR1-F | AGTGTAGATTGATGGATGGT |
| OsNR1-R | CAGTCGTAGTAATATCTCGGT |
| OsNR2-F | CGAACCTGGAGAAGATGA |
| OsNR2-R | TTTCTAATTGGCACGACAC |
